# Surface Dynamics in Allosteric Regulation of Protein-Protein Interactions: Modulation of Calmodulin Functions by Ca^2+^

**DOI:** 10.1371/journal.pcbi.1003028

**Published:** 2013-04-04

**Authors:** Yosef Y. Kuttner, Tal Nagar, Stanislav Engel

**Affiliations:** 1 Bioinformatics Core Facility, National Institute for Biotechnology in the Negev, Ben-Gurion University of the Negev, Beer-Sheva, Israel; 2 Department of Clinical Biochemistry and Pharmacology, National Institute for Biotechnology in the Negev, Ben-Gurion University of the Negev, Beer-Sheva, Israel; University of Houston, United States of America

## Abstract

Knowledge of the structural basis of protein-protein interactions (PPI) is of fundamental importance for understanding the organization and functioning of biological networks and advancing the design of therapeutics which target PPI. Allosteric modulators play an important role in regulating such interactions by binding at site(s) orthogonal to the complex interface and altering the protein's propensity for complex formation. In this work, we apply an approach recently developed by us for analyzing protein surfaces based on steered molecular dynamics simulation (SMD) to the study of the dynamic properties of functionally distinct conformations of a model protein, calmodulin (CaM), whose ability to interact with target proteins is regulated by the presence of the allosteric modulator Ca^2+^. Calmodulin is a regulatory protein that acts as an intracellular Ca^2+^ sensor to control a wide variety of cellular processes. We demonstrate that SMD analysis is capable of pinpointing CaM surfaces implicated in the recognition of both the allosteric modulator Ca^2+^ and target proteins. Our analysis of changes in the dynamic properties of the CaM backbone elicited by Ca^2+^ binding yielded new insights into the molecular mechanism of allosteric regulation of CaM-target interactions.

## Author Summary

Protein-protein interactions (PPI) play an essential role in virtually all physiological processes. Knowledge of the principles governing PPI is of fundamental importance for understanding the organization and functioning of biological systems. Furthermore, a number of human diseases intractable to conventional therapies are caused by aberrant PPI, and an ability to control these interactions could help pave the way to the development of novel, more efficient treatment strategies. The present research was undertaken in the hope of shedding new light on the process of PPI regulation by small-molecule compounds known as allosteric modulators. These modulators bind at sites distinct from those directly involved in PPI and affect the ability of proteins to form complexes. Employing a recently developed computational approach for the analysis of protein surfaces, we explored the effects of binding by a modulator, the Ca^2+^-ion, on the dynamic properties of the model protein calmodulin (CaM). CaM is a Ca^2+^ sensing protein responsible for the regulation of a variety of physiological processes. The analysis enabled us to identify CaM surfaces involved in bimolecular recognition and yielded new insights into the molecular mechanism of PPI regulation by allosteric modulators.

## Introduction

Elucidating the basic principles governing molecular recognition in biological systems is important for advancing our understanding of the organization and functioning of biological networks, both metabolic and signaling, and hence for the development of pharmacological tools for the correction of aberrant disease-causing interactions. A growing body of evidence supports a role for the dynamic properties of protein surfaces in bimolecular recognition [1], [2], [3], [4]. We have recently reported on a novel computational approach employing steered molecular dynamics simulation (SMD) for analysis of the dynamics of protein surfaces [5]. We demonstrated, in a number of model proteins, the existence of a distinct pattern whereby surface residues whose backbone is resistant to perturbations introduced by an external force tend to cluster, giving rise to so-called “stability patches” surrounded by areas of moderate to high backbone compliance. These “stability patches” have a propensity to localize in the surface areas implicated in bimolecular recognition [5].

In the present work, we employed SMD analysis to investigate the paradigm of regulation of protein-protein interactions by allosteric modulators. Allosteric modulators bind at a site orthogonal to the protein-protein interface, inducing conformational changes that affect the proteins' propensity to form complexes. As a model system we used the regulation of calmodulin (CaM) interaction with target proteins by the allosteric modulator Ca^2+^. CaM is a ubiquitous eukaryotic protein that regulates various cellular functions through Ca^2+^-dependent interaction with a diversity of target proteins, such as protein kinases, phosphatases, ion channels, and the elements of the cytoskeleton [6]. CaM is composed of two globular domains (N-lobe and C-lobe) separated by a long helical segment. Each lobe contains a pair of EF-hand helix-loop-helix motifs (designated EF-1 to EF-4 starting from the N-terminus). EF-hand motifs are structural elements accountable for Ca^2+^ coordination and occur widely in the family of calcium-binding proteins. In CaM, the Ca^2+^-binding loops of the EF-hands span a linear sequence of 12 amino acids, six of which provide Ca^2+^ ligands (positions 1, 3, 5, 7, 9 and 12 in the linear sequence, Supplementary Figure S1) [7], [8], [9], [10].

The lobes in apo- (Ca^2+^-free) and Ca^2+^-loaded CaM share similar elements of secondary structure. However, in apo-CaM the four α-helices of the lobe's two EF-hands form a characteristic bundle structure (“closed” conformation), whereas in the Ca^2+^-loaded state the helical bundle splits, revealing a hydrophobic cleft (“open” conformation) [11], [12]; this cleft plays an essential role in CaM interaction with target proteins [13], [14], [15]. Due to the flexibility of the central linker, both N- and C-lobes can contribute to target binding. In the “open” conformation, residues in the hydrophobic cleft interact with the anchoring residues in the amphipathic α-helix of the CaM-binding domain in target proteins. The CaM-binding domains in various target proteins share low sequence similarity, and the mechanism accountable for CaM's remarkable ability to recognize structurally diverse proteins is not completely understood [16].

Hypothesizing a correlation between the dynamic properties of the backbone of surface-exposed residues and the functional characteristics of the corresponding surface areas, we carried out a comparative SMD analysis of CaM structure in its “closed” (apo-) and “open” (Ca^2+^-loaded) conformations. The results demonstrate that Ca^2+^ binding leads to a major redistribution of surface residues on the basis of their “resistance coefficients”, a parameter we devised to quantify backbone resistance to local structural perturbations induced by an externally applied force [5]. This redistribution appears to correspond to the changes in the functional properties of the CaM surfaces, *i.e.* the ability to interact with target proteins. This study offers new insights into the molecular mechanism of regulation of CaM interaction with target proteins by the allosteric modulator Ca^2+^.

## Methods

### Preparation of structures

In this study we used solution structures of the apo- (PDB: 1F54) and Ca^2+^-loaded (PDB: 1F55) N-terminal domain of yeast calmodulin (the first structure in the assembly of the lowest energy conformers) [17]. The structures were solvated in a water box (57×56×53 Å^3^ [16,000 atoms in total] for the apo-N-lobe and 62×51×53 Å^3^ [16,034 atoms in total] for the Ca^2+^-loaded N-lobe), energetically minimized and equilibrated by molecular dynamics with a stepwise release of spatial constraints, applied first to the entire protein (100 ps), then to the backbone atoms only (100 ps). The equilibration of the yCaM mutants' structures was carried out without structural constraints for an additional 100 ps. These and subsequent simulations were carried out at 310K using the NAMD program (version 2.8b3) [18] and the CHARMM(31) force field for proteins [19].

### Steered molecular dynamics simulation (SMD)

The equilibrated structures were used to generate a list of surface-exposed residues, defined as residues with a solvent accessible surface area (SASA) greater than 30 Å^2^ (57 residues for the apo-N-lobe [Supplementary Figure S2] and 59 residues for the Ca^2+^-loaded N-lobe [Supplementary Figure S3]). For each surface residue, SMD was carried out without energy minimization using the Cα atom as SMD atom. The spring constant of a virtual spring connecting the SMD and dummy atoms was set to 7 kcal/mol/Å^2^. The dummy atom was pulled at a *constant* velocity (0.15 Å/ps) in the direction of the vector connecting the center of mass of a 4 Å hemisphere surrounding the SMD atom and the SMD atom (a direction approximating a normal to the local surface) [5]. During the simulation, all atoms within 13 Å of the SMD atom were set free to move, whereas the Cα atoms outside the resulting 13 Å “free” hemisphere were kept fixed. Periodic boundary conditions were used, and the constant pressure method was employed to control the periodic cell fluctuations. To calculate electrostatic interactions, the particle mesh Ewald method implemented in NAMD was used. For each residue, SMD was carried out at least twelve times and plots of applied forces versus distances traveled by the dummy atoms were generated from the SMD trajectories. The plots were analyzed by a linear regression using the Prism 6 program (GraphPad Software, Inc.), and the slopes were referred to as the “resistance coefficients” [5]. The analysis was limited to a distance of 2.5 Å traveled by the dummy atoms, within the range of nonbonded interactions, to prevent protein unfolding due to the pulling to probe the dynamics of Cα atoms in the vicinity of their equilibrium position in the structure. In this range, plots of applied forces versus distances traveled by the dummy atoms exhibit linearity [5].

### Equilibrium molecular dynamics simulation (EMD)

The structures of the apo- and Ca^2+^-loaded N-lobes prepared as described above were further equilibrated at 310 K for 1 ns without spatial constraints, followed by an additional 1 ns simulation to collect RMSD data. For each structure, EMD was repeated three times and the mean RMSD values for individual residues over the time of trajectory were calculated using the RMSD module implemented in the VMD program [20]. The correlation coefficients between the RMSD and “resistance coefficient” data sets were calculated using the CORREL statistical function implemented in the Excel program.

The KFC (Knowledge-based FADE and Contacts) server [21] was used to delineate protein-protein interfaces. Structure visualization and image generation were carried out using the VMD program [20]. Intel Core i7 CPU computers were used for the calculations.

## Results

As model protein in this study we used the isolated N-lobe of the yeast *S. cerevisiae* CaM (yCaM), whose experimental structures are available for both apo- and Ca^2+^-loaded states (PDB: 1F54 and 1F55) [17], [22]. The conformations assumed by the isolated yCaM N-lobe in apo- and Ca^2+^-loaded states appear to be almost identical to those of the N-terminal domain of vertebrate CaM in the corresponding states [17]. The structures of the isolated N-lobes represent the end-points of the process of allosteric transition induced by Ca^2+^-binding and are devoid of the effects of inter-lobe interactions characteristic of full length calmodulins.

### Resistance coefficients

**Figure 1:**
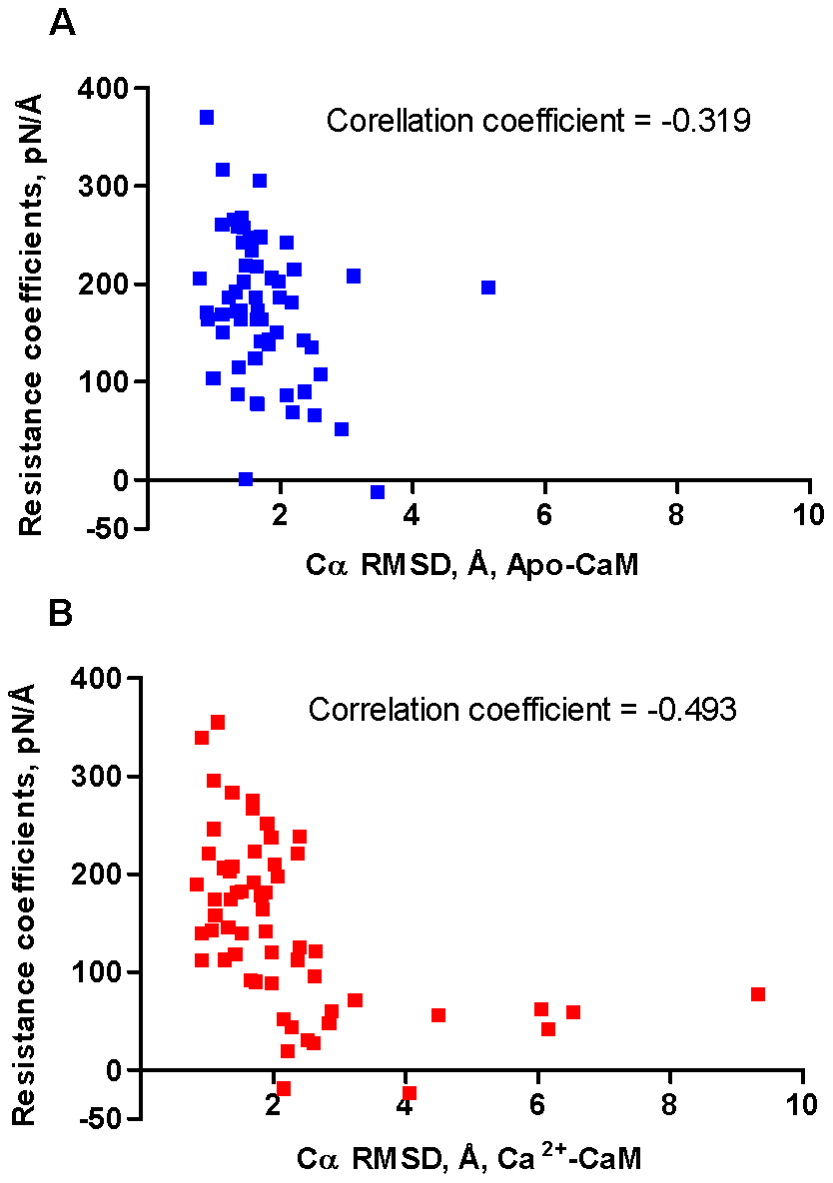
Correlation between the “resistance coefficients” and RMSD values. The “resistance coefficients” and RMSD of Cα atoms of surface-exposed residues in the apo-, **A**, and Ca^2+^-loaded, **B**, yCaM N-lobe were calculated using SMD and EMD analysis, respectively, as described in the Methods. For the correlation analysis, mean values of the “resistance coefficients” and RMSD obtained from multiple rounds of simulations (see Methods) were used. The correlation coefficients between the two data sets were calculated using the CORREL statistical function implemented in the Excel program.

In equilibrium molecular dynamics simulation (EMD) sampling of protein conformations takes place in the vicinity of the thermal equilibrium state. Ligand binding, however, may induce local conformational changes whose amplitude exceeds that characteristic of thermal fluctuations. Consequently, EMD may fail to simulate such processes adequately. In SMD, however, external forces applied at a selected atom (or group of atoms) can produce local perturbations proceeding beyond thermal fluctuations and thus enable sampling of conformational states inaccessible by EMD. We had previously hypothesized that the propensity of the backbone of surface-exposed residues to comply with the steering forces exercised by incoming ligands may facilitate induced fit and shaping of a ligand-binding cavity, thereby maximizing exploitation of the available interaction potential [5]. To quantify the extent of backbone compliance, we devised a semi-quantitative parameter called the “resistance coefficient”, which represents the slope of a linear plot relating the pulling force applied to the SMD atom (Cα) and the distance travelled by the dummy atom (see Methods). We showed that the surface regions known as energetic “hot-spots” exhibit a characteristic pattern consisting of a “stability patch” formed by residues with high “resistance coefficients” surrounded by areas of relatively high backbone compliance [5]. In the present work, we further explored the nature of the “resistance coefficients” by comparing them with the atomic fluctuations (RMSD) of the corresponding Cα atoms obtained from an unbiased EMD. We demonstrated that there is a limited degree of negative correlation between the “resistance coefficients” and the RMSD values calculated for both apo- (correlation coefficient −0.319) and Ca^2+^-loaded (correlation coefficient −0.493) N-lobe structures (Figure 1A and B). This result indicates that the thermal fluctuation of the backbone atoms exerts a certain, though limited, influence on the values of the “resistance coefficients”. However, other factors, most probably linked to the differential capacity of local structures to comply with external forces, appear to exert a strong modulatory effect. It is noteworthy that the highest variance in the values of the “resistance coefficients” is observed within a narrow range of RMSD values (Figure 1A and B). It appears that the “resistance coefficients” are capable of capturing unique aspects of backbone dynamics that could not be described by the analysis of thermal fluctuations alone. Below we will use the term “mobility” to address backbone dynamic properties quantified in terms of the “resistance coefficients”.

### Apo-yCaM N-lobe

**Figure 2:**
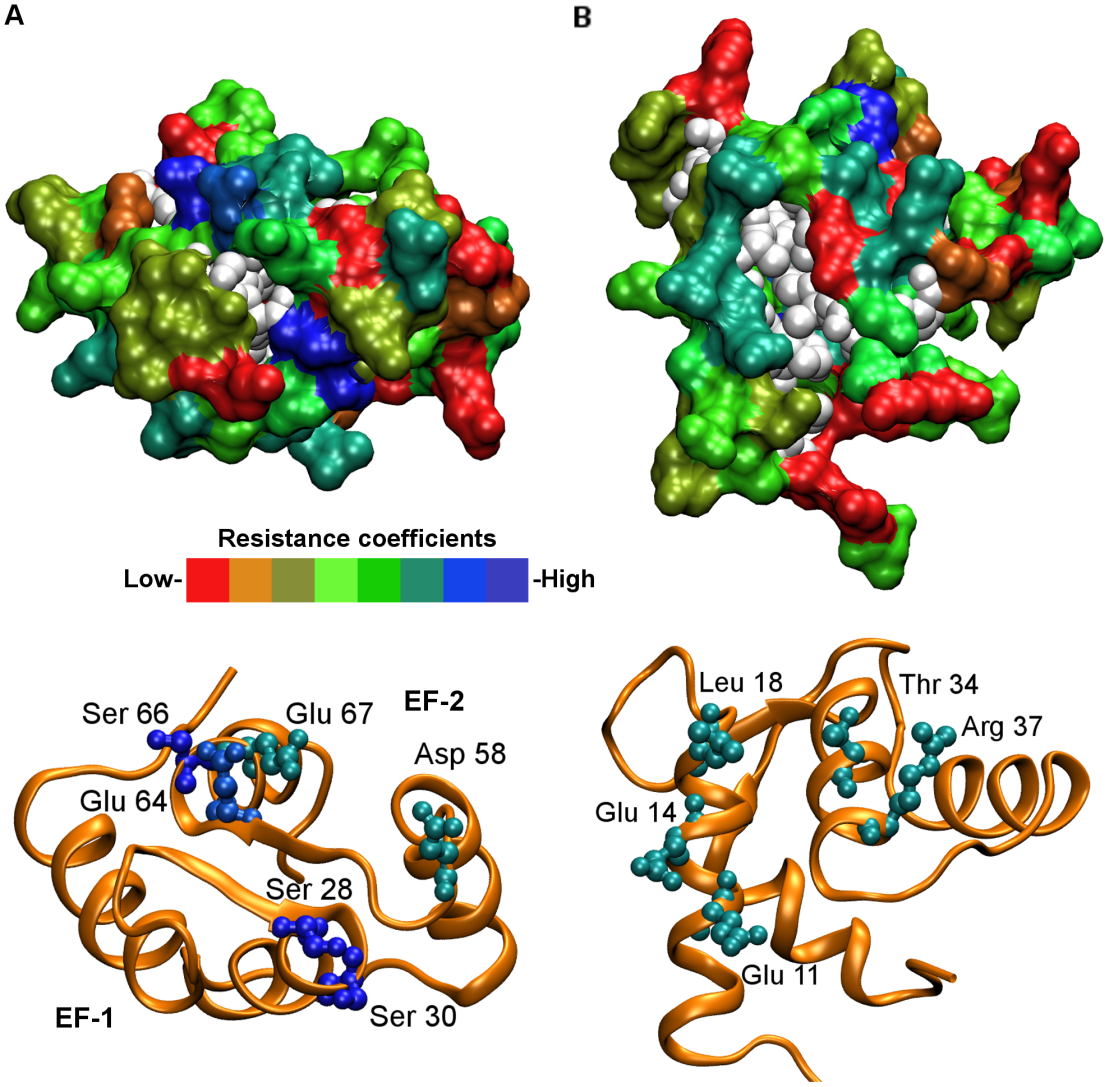
SMD analysis of apo-yCaM N-lobe. The surface of the apo-yCaM N-lobe (“closed” conformation) (PDB: 1F54) [17] is oriented so that the Ca^2+^ binding loops face the viewer, to demonstrate the position of the discontinuous “stability patch” associated with the two Ca^2+^-binding sites (in EF-1 and EF-2), **A**; rotated by 90° around a horizontal axis, to demonstrate the location of the “stability patch” associated with the EF-1 entering and exiting α-helices, **B**. On the upper images, surface-exposed residues are shown in the “Surf” representation [20] and colored according to the corresponding “resistance coefficients”. Red and blue in the color scale correspond to the residues with the lowest (<100 pN/Å) and highest (>300 pN/Å) resistance coefficients, respectively. Residues that are not surface-exposed in the apo-yCaM N-lobe (SASA<30 Å) are shown in the VDW representation and colored in white (not subjected to the SMD). The bottom images show identically oriented N-lobes in the cartoon representation (orange) with the residues constituting the “stability patches” shown in the CPK representation [20], colored according to the corresponding “resistance coefficients” and labeled.

The apo-yCaM N-lobe adopts a typical “closed” conformation, in which the hydrophobic residues at the helical bundle core are shielded from the solvent (PDB: 1F55) [17]. The SMD surface analysis demonstrated a moderate overall mobility of the backbone (Figure 2 and Supplementary Figure S2) with highly immobile residues localized in two distinct surface areas. The first area is associated with the Ca^2+^-binding loops: positions 9 and 11 (Ser 28 and Ser 30) in EF-1 and positions 3, 9, 11 and 12 (Asp 58, Glu 64, Ser 66 and Glu 67) in EF-2 are occupied by static residues (Figure 2A and Supplementary Figure S1). Within the EF-hand motifs, the turn of the loop brings the static residues into close proximity, resulting in the formation of a discontinuous “stability patch” associated with the two Ca^2+^-binding sites.

**Figure 3:**
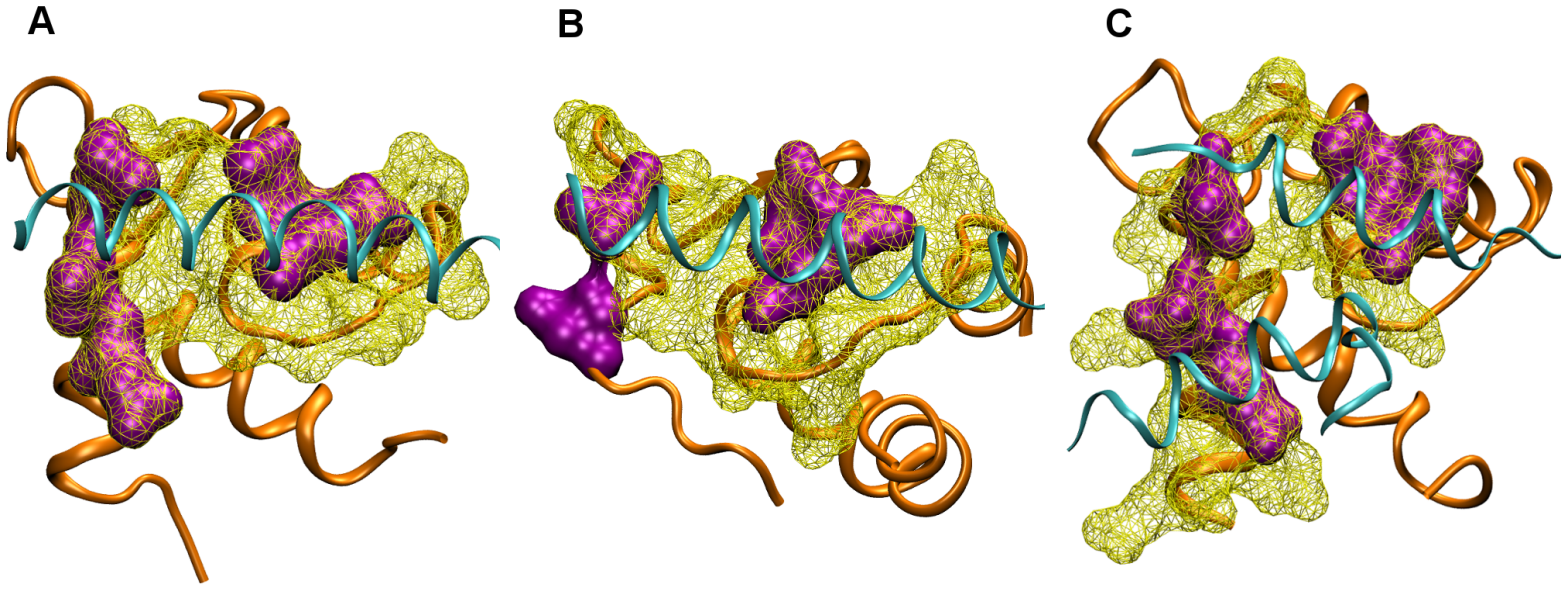
Complexes of Ca^2+^-free EF-hand proteins with targets. The complex of mouse apo-CaM with the IQ-1 domain of unconventional myosin V (PDB: 2IX7) [28], **A**. The complex of yeast Mlc1p (myosin light chain) with the IQ-2 domain of Myo2p (myosin V) (PDB: 1M45) [23], [26], [29]; Mlc1p is a CaM-like EF-hand protein that does not bind Ca^2+^, **B**. The complex of human apo-CaM with the adenylyl cyclase domain of anthrax edema factor (PDB: 1K93) [27], [41], **C**. The EF- hand and target proteins are shown in the cartoon representation and colored in orange and cyan, respectively. For clarity, only elements of the structure in direct contact with EF-hand proteins are shown for the target proteins. CaM residues at the complex interfaces are shown in the Surf representation (wireframe) and colored in yellow. Amino acids corresponding to the apo-yCaM “stability patch” residues Glu 11, Glu 14, Leu 18, Thr 34 and Arg 37 are shown in the “Surf” representation and colored in magenta.

The second static area comprises residues of the entering (at the N-terminal end) and exiting (at the C-terminal end) α-helices of EF-1 (Glu 11, Glu 14, Leu 18, Thr 34 and Arg 37) (Figure 2B), whose mobility is slightly higher (resistance coefficients 230–270 pN/Å) than that of the residues in the “stability patch” associated with the Ca^2+^-binding loops (270–360 pN/Å). In their Ca^2+^-free form, CaM and CaM-like EF-hand proteins are capable of interacting with a variety of target proteins, where their role appears to be regulatory [23], [24], [25], [26], [27]. Structural data reveal that in the resulting complexes, the N-lobe assumes a typical “closed” conformation, and its interaction with the target is mediated by mostly hydrophilic residues of the EF-1 entering and exiting α-helices [26], [27], [28]. It is noteworthy that residues constituting the “stability patch” associated with the EF-1 α-helixes in apo-yCaM are conserved throughout the CaM family and appear to localize at the interaction interface in the aforementioned complexes (Figures 2B and 3). For instance, mouse CaM counterparts of the apo-yCaM “stability patch” residues Glu 14, Leu 18 and Arg 37 interact with the residues of the IQ motif in the CaM complex with myosin V heavy chain (PDB: 2IX7) [28]. According to the experimental data, the contribution of the N-lobe “closed” conformation to the affinity of the CaM-target complexes is low [23], [28], [29], [30]. This observation is consistent with the results of the SMD analysis demonstrating that the “stability patch” at the complex interface consists of residues whose resistance coefficients are comparatively low (230–270 pN/Å), a feature that may affect the association kinetics of the complex formation [5].

### Ca^2^-loaded yCaM

**Figure 4:**
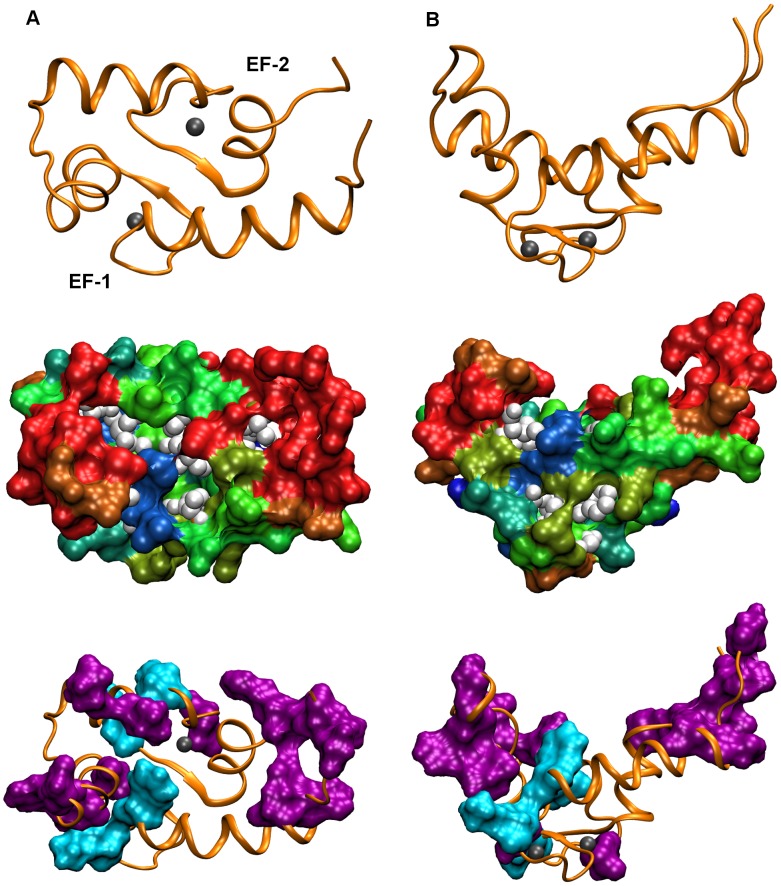
SMD analysis of Ca^2+^-loaded yCaM N-lobe. The surface of the Ca^2+^-loaded yCaM N-lobe (“open” conformation) (PDB: 1F55) [17] is oriented: so that the hydrophobic cleft faces the viewer, **A**; so that, after rotation by 90° around a horizontal axis, the plane of the EF-1 α-helices faces the viewer, **B**. The upper images show the N-lobe in the cartoon representation. Ca^2+^ ions are shown as grey spheres. The middle images show two identically oriented N-lobes in the “Surf” representation colored according to the “resistance coefficients” with the same color assignments as in Figure 2. The bottom images demonstrate two identically oriented N-lobes with surface-exposed residues whose “stability coefficients” increased (in cyan) or decreased (in purple) by more than 70 pN/Å as a result of Ca^2+^ binding shown in the “Surf” representation.

Ca^2+^ binding induces gross conformational changes in the structure of the yCaM N-lobe [17], leading to redistribution of surface-exposed residues according to their resistance coefficients (Figure 4 and Supplementary Figure S3 and S4). Separation of the EF-hands α-helices exposes a hydrophobic cleft to which a variety of target proteins bind. The transition from “closed” to “open” conformation results in increased overall backbone mobility. Figure 4 (bottom) highlights surface residues whose resistance coefficients are affected by Ca^2+^ binding. Residues whose mobility increases (in purple) are primarily associated with the separating helices, whereas those with a decreased mobility (in cyan) tend to localize at the base of the hydrophobic cleft. The consequence of this rearrangement is the disappearance of the “stability patch” associated with the EF-1 α-helixes and the emergence of a new “stability patch” at the bottom of the hydrophobic cleft comprising Leu 18, Phe 19, Asp 50 and Ala 46 (Figure 4), residues that are conserved throughout the CaM family.

**Figure 5:**
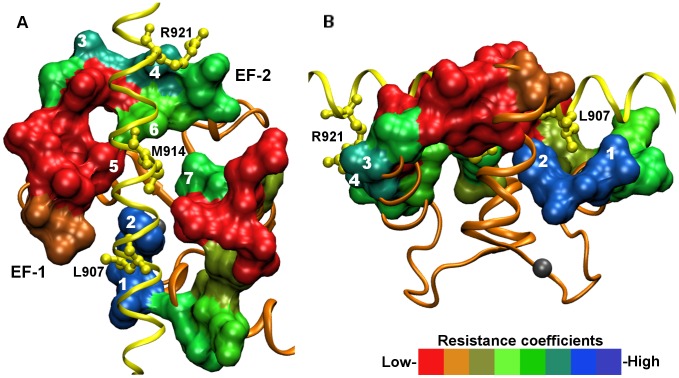
CaM complex with target protein. The N-lobe of CaM from the yeast *K. lactis* (in orange) is shown in a complex with the spindle pole body protein Spc110 from the yeast *S. cerevisiae* (in yellow) (PDB: 4DS7). CaM residues at the interaction interface are shown in the “Surf” representation and colored according to the “resistance coefficients” calculated for the Ca^2+^-loaded yCaM N-lobe, with the same color assignments as in Figure 2. The Spc110 “anchoring” residues are shown in the “ball-and-stick” representation (in yellow) and labeled. Numbers 1, 2, 3 and 4 denote amino acids corresponding to the “stability patch” residues Leu 18, Phe 19, Ala 46 and Asp 50, respectively, at the bottom of the yCaM hydrophobic cleft. Numbers 5, 6 and 7 denote amino acids corresponding to the mobile residues Met 36, Leu 51 and Leu 71, respectively, which form the binding site for the Spc110 “anchoring” residue Met 914. The CaM surface is oriented so that the hydrophobic cleft faces the viewer, **A**; rotated by 90° around a horizontal axis and counterclockwise, **B**.

We assigned the resistance coefficients calculated for the residues in Ca^2+^-loaded yCaM (*S. cerevisiae*) to the corresponding residues in CaM from the related yeast *Kluyveromyces lactis*, which shares 95% sequence identity with yCaM and whose experimental structure in the complex with target protein Spc110, a spindle pole body protein from *S. cerevisiae*, is available (PDB: 4DS7). We demonstrated that the “stability patch” residues Leu 18 and Phe 19 at the bottom of the hydrophobic cleft delineate a binding pocket for Leu 907, the anchoring residue of Spc110 (Figure 5). Asp 50, an immobile residue on the opposite side of the hydrophobic cleft, forms an electrostatic interaction with Spc110 residue Arg 921. In the middle of the hydrophobic cleft, there is a binding pocket for another Spc110 anchoring residue Met 914, composed of mobile residues Met 36, Leu 51 and Leu 71 (marked 5, 6 and 7 in Figure 5). The anchoring residues Leu 907 and Met 914 are elements of the so-called CaM-recognition sequence, an attribute of the amphipathic α-helix of a typical CaM-binding domain [31], [32], [33]. Different yCaM target proteins may have different distances between anchoring residues. For instance, in Spc110 the anchoring residues are separated by six amino acids (Leu 907 and Met 914), whereas in calcineurin, another yCaM target protein, the number of separating residues is five (Ile 465 and Met 471) [33]. The ability of yCaM to accommodate target proteins with differently separated anchoring residues suggests a substantial degree of structural flexibility in the corresponding docking sites. This inference is consistent with our observation that the docking site in the middle of the hydrophobic cleft is formed of mobile residues (Met 36, Leu 51 and Leu 71). This feature may facilitate structural adaptation at the complex interface and, in combination with the highly mobile residues constituting the side walls of the hydrophobic cleft (Figure 5), may contribute to the ability of CaM to recognize structurally diverse target proteins.

Being immobile in both the apo- and the Ca^2+^-loaded states, in the “closed” yCaM conformation Leu 18 is a part of the “stability patch” associated with the EF-1 entering and exiting α-helices. Transition to the “open” conformation results in “melting” of this “stability patch” and shifts Leu 18 into a new position facing the hydrophobic cleft. At the bottom of the hydrophobic cleft Leu 18 takes part in the formation of an alternative “stability patch” corresponding to the binding pocket for the anchoring residue of the CaM recognition sequence (Figure 5).

In contrast to Leu 18, whose mobility is largely unaffected by Ca^2+^ binding, the backbone compliance of Phe 19 is a function of yCaM Ca^2+^ occupancy. While mobile in the “closed” yCaM conformation, in the “open” conformation Phe 19, along with Leu 18, forms the “stability patch” at the bottom of the hydrophobic cleft. The Phe 19 residue is highly conserved throughout the CaM family, and its substitution by Ala has been shown to profoundly affect yCaM's ability to bind and activate target proteins [34]. Elucidation of the structural basis underpinning the regulation of Phe 19 dynamics by Ca^2+^ may be expected to shed valuable light on the molecular mechanism of CaM allosteric transitions.

### Structural basis of Phe 19 dynamics

**Figure 6:**
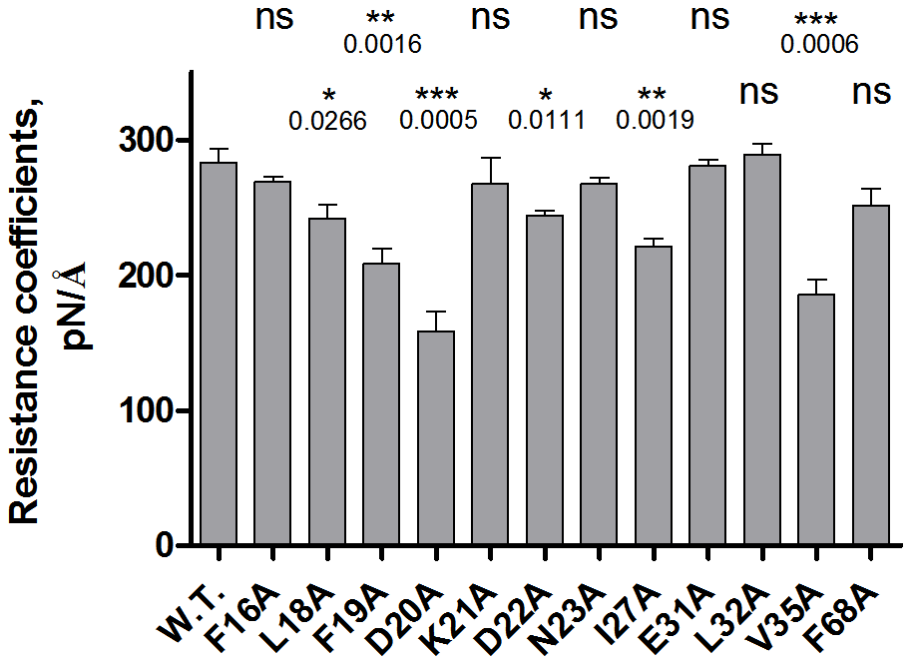
*In silico* mutagenesis analysis of Phe 19-adjoining residues. The Phe 19 “resistance coefficients” were calculated in the structures of *in silico* point mutants of the Ca^2+^-loaded yCaM N-lobe with residues in the immediate vicinity of Phe 19 replaced by Ala. For each mutant, SMD analysis was carried out at least 12 times. The error bars represent the standard error of the mean. A two-tailed unpaired *t*-test was used to assess the significance of the difference in the means observed between the WT and mutant proteins. *P* values are indicated for the sets with statistically significant difference (*ns* - not significant).

To elucidate the mechanism of Phe 19 transition from a mobile to a static state under the influence of Ca^2+^, we explored the role of nearby residues in restricting the mobility of the Phe 19 backbone in the “open” conformation. The structure of the Ca^2+^-loaded yCaM N-lobe was used to generate a series of *in silico* point mutations in which residues in the immediate vicinity of Phe 19 were replaced by alanine, after which the effect of these substitutions on the resistance coefficient of Phe 19 was evaluated. In several mutants a significant destabilization of the Phe 19 backbone was observed (Figure 6). The substitution of the flanking bulky branched-chain amino acids Ile 27, Val 35 and Leu 18 by alanine enhanced mobility of the Phe 19 backbone. A decreased resistance coefficient was also observed in the Phe19Ala mutant, demonstrating that the nature of the side-chain is an important factor in determining the mobility of the residue's backbone. The most profound effect on the resistance coefficient of Phe 19, however, was observed for replacement by alanine of Asp 20, a highly conserved residue at position 1 of the EF-1 Ca^2+^-binding loop (Supplementary Figure S1) [25], [35], essentially reversing the stabilizing effect of Ca^2+^ binding. The surface exposed Phe 19 is located in an unstructured region at the beginning of the Ca^2+^-binding loop, and Ca^2+^ coordination mediated by the adjacent Asp 20 may offer a stabilizing effect on the dynamics of the Phe 19 backbone.

## Discussion

In a recent study we had hypothesized that the presence of “stability patches” at the interface of protein-protein complexes may facilitate bimolecular recognition by decreasing the energetic cost of initial association and providing an “anchor” for nucleating the directional process of structural adaptation at the interface required for an efficient utilization of the available interaction potential [5]. We demonstrated that there exists a correlation between the locations of the known energetic “hot spots” and the “stability patches” at the interaction interfaces. We also hypothesized that small-molecule ligands targeting protein-protein interfaces may exploit the unique structural organization of the “hot spots” to secure a binding energy sufficient for the formation of high-affinity complexes [5]. In the present study we employed SMD surface analysis to acquire new insights into the mechanism of modulation of protein-protein recognition by allosteric ligands. Using the regulation of CaM - target interactions by Ca^2+^ as a model system, we explored the process of Ca^2+^-induced CaM transition from the “closed” to “open” conformation by analyzing changes in the dynamic properties of surface-exposed residues associated with the transition.

The SMD surface analysis revealed the presence of two discontinuous “stability patches” in the “closed” conformation of the yCaM N-lobe (Figure 2A and B). The first “stability patch” is associated with the Ca^2+^-binding loops. The two loops are not identical in the number and location of the static residues: positions 9 and 11 in EF-1, and positions 3, 9, 11 and 12 in EF-2, are occupied by highly immobile residues (Figure 2A and Supplementary Figure S1). Ca^2+^ binding to the yCaM N-lobe has been shown to be a highly cooperative process [8]. The resolution of the analytical methods used, however, did not allow the relative order of titration of the EF-hands by Ca^2+^ to be determined. Assuming that static residues play a role in facilitating initial protein-ligand association [5], the presence of a higher number of immobile residues in EF-2 suggests that this domain may be the first to bind Ca^2+^.

In the final complex, residues at positions 3, 9 and 12 participate in Ca^2+^ coordination [7]. These residues are functionally conserved throughout the CaM family. In contrast, position 11 is not conserved and is occupied by Lys (EF-1) and Pro (EF-2) in vertebrate CaMs, whereas in the CaMs of several ascomycota fungi (including *Saccharomyces cerevisiae*, *Kluyveromyces lactis*, *Schizosaccharomyces pombe* and *Ashbya gossypii*), Ser, Thr or Asn are found at the corresponding sites. As demonstrated in *S. cerevisiae* and *S. pombe*, in yeast CaMs the N-lobe affinity for Ca^2+^ is significantly higher than that of vertebrate CaMs [8], [36], [37]. In contrast to vertebrate CaMs, residues at position 11 of the Ca^2+^-binding loops in the N-lobe of yeast CaMs possess a potential for Ca^2+^-coordination. Their highly immobile character suggests a potential role for these solvent-exposed residues in the early steps of Ca^2+^ ligation, *e.g.* by facilitating Ca^2+^ extraction from the bulk solution.

The CaM-binding sequences of target proteins do not exhibit broad consensus. However, a large group of proteins that includes most unconventional myosins, ion channels, small GTPases, IQGAP and neuronal proteins bind CaM through the so-called IQ domains, which share a consensus IQxxxRGxxxR motif [23], [24], [25], [26]. CaM and CaM-like proteins such as myosin light chains can maintain their interaction with the IQ domains in the presence or absence of Ca^2+^ [23], [26], [29]. The Ca^2+^-free C-lobe interacting with the IQxxxR portion of the IQ motif adopts a so-called “semi-open” conformation with only a partial exposure of the hydrophobic core. In contrast, the Ca^2+^-free N-lobe interacting with the GxxxR portion of the IQ motif assumes a typical “closed” conformation [26], [28]. Although the precise complex geometry is a function of the identity of variable residues in the GxxxR motif (in some cases the N-lobe can remain free in solution, potentially mediating the formation of ternary complexes with other proteins [26], [38], [39], [40]), interaction generally involves the N-lobe surfaces encompassing the EF-1 entering and exiting α-helices [23], [26], [28]. Interestingly, human Ca^2+^-free CaM deploys similar surface elements to engage the oedema factor, a calmodulin-activated adenylyl cyclase domain of anthrax exotoxin, in a process crucial for anthrax pathogenesis [27], [41]. The SMD analysis of the “closed” N-lobe conformation revealed the presence of an extended discontinuous “stability patch” whose location overlaps with the interface in the aforementioned complexes (Figures 2B and 3).

CaM is a mediator of the regulation of unconventional myosin V activity by Ca^2+^. The dependence of myosin activity on Ca^2+^ concentration has been interpreted in terms of a decreased affinity and subsequent dissociation of the IQ domains from CaM in the course of its transition from the apo- to the Ca^2+^-loaded state [28], [29], [42]. This interpretation is consistent with our observation that the “stability patch” associated with the EF-1 α-helices “melts” as a result of the N-lobe transition into the “open” conformation. Interestingly, unlike other residues constituting this “stability patch”, whose backbone becomes destabilized upon separation of the helices, Leu 18 maintains its highly immobile character. Instead, in the “open” conformation, its position shifts to face the hydrophobic cleft, where it takes part in the formation of an alternative “stability patch”.

The transition from the “closed” to the “open” conformation is manifested in separation of the helices and emergence of the hydrophobic cleft. At its base the hydrophobic cleft harbors a cluster of highly immobile residues, constituting the “stability patch” whose presence may facilitate initial CaM association with target proteins [5]. The “stability patch” residues Leu 18 and Phe 19 delineate a cavity to which the anchoring residue of the CaM-recognition sequence binds. Residue Leu 18, whose position shifts in a Ca^2+^-dependent manner between the two alternative “stability patches”, may be acting in this context as the element of a toggle switch mechanism facilitating the formation of yCaM complexes with alternative targets specific either to the “closed” or to the “open” yCaM conformations.

In contrast, Phe 19, a mobile residue in the “closed” apo-yCaM conformation, becomes distinctly stabilized upon Ca^2+^ binding. *In silico* mutagenesis analysis of residues adjoining Phe 19 in the structure of the Ca^2+^-loaded N-lobe has identified Asp 20 as a residue crucial for maintaining Phe 19's static character. Asp 20 is a highly conserved Ca^2+^-coordinating residue at position 1 of the Ca^2+^-binding loop and is indispensable for Ca^2+^ binding [25], [35]. Our results suggest an additional role for this residue, namely as enabler of an allosteric coupling between Ca^2+^ binding and the formation of the “stability patch” at the bottom of the hydrophobic cleft, a process that results in CaM conversion into a state competent of productive interaction with a variety of target proteins.

The phenyl ring of Phe 19 appears to be an important determinant of the mobility of this residue's backbone. Experimental identification of energetic “hot spots” relies on replacement of interfacial residues by alanine, followed by determination of the effects of this substitution on the free energy of complex formation [43]. We hypothesized that the large drop in free binding energy associated with substitution of a “hot spot” residue could be due to the combined effect of, first, loss of the specific interactions mediated by the side-chain and, second, increased mobility of the residue's backbone.

We had previously concluded that a typical “hot spot” region would include an area of low mobility facilitating initial association of the interacting partners, and a region of high mobility allowing mutual structural adaptation at the interface [5]. Viewed from such a perspective, it seems that the hydrophobic cleft of the “open” yCaM conformation exhibits the necessary characteristics of a typical “hot spot” region, with compliance properties ideally suited to supporting CaM interaction with a variety of structurally diverse proteins.

In conclusion, the present study demonstrates the ability of SMD analysis to pinpoint elements of the CaM surface implicated in bimolecular recognition. A comparative analysis of the distribution of surface-exposed residues according to their backbone resistance coefficients in different conformational states may offer new insights into the molecular mechanism of allosteric transition and regulation of protein function by allosteric modulators.

## Supporting Information

Figure S1 **Sequence of Ca^2+^-binding loops in yCaM N-lobe.** The 1–12 numbering corresponds to the amino acid positions within the linear sequence of the putative Ca^2+^-binding loops. Residues that provide Ca^2+^ ligands are shaded.
(TIFF)

Figure S2 **Resistance coefficients of surface-exposed residues in apo-yCaM.** Surface-exposed residues in the structure of the apo-yCaM N-lobe (PDB: 1F54) were subjected to SMD analysis and the “resistance coefficients” calculated as described in the Methods. For each residue SMD analysis was carried out at least 12 times. The error bars represent the standard error of the mean. Color assignments are according the residues' “resistance coefficients” as described in the legend to Figure 2.
(TIF)

Figure S3 **Resistance coefficients of surface-exposed residues in Ca^2+^-loaded yCaM.** Surface-exposed residues in the structure of the Ca^2+^-loaded yCaM N-lobe (PDB: 1F55) were subjected to SMD analysis. Other details as in the legend to Supplementary Figure S2.
(TIF)

Figure S4 **Ca^2+^-induced changes in resistance coefficients of surface-exposed residues.** The “resistance coefficients” of surface-exposed residues in the structures of both apo- and Ca^2+^-loaded yCaM N-lobe were used to calculate the changes. Positive values correspond to residues whose stability increased as a result of Ca^2+^ binding, whereas negative values correspond to residues with increased mobility. Residues whose resistance coefficients increased or decreased by more than 70 pN/Å are colored in cyan or purple, respectively. In Figure 4 (bottom), the same residues are shown in the Surf representation and colored similarly.
(TIF)
