# Supplementary figures and images for: Surface Dynamics in Allosteric Regulation of Protein-Protein Interactions: Modulation of Calmodulin Functions by Ca2+
Source: PLoS Comput Biol. 2013 Apr 4;9(4):e1003028. doi: 10.1371/journal.pcbi.1003028 (PMC3617199; doi:10.1371/journal.pcbi.1003028)

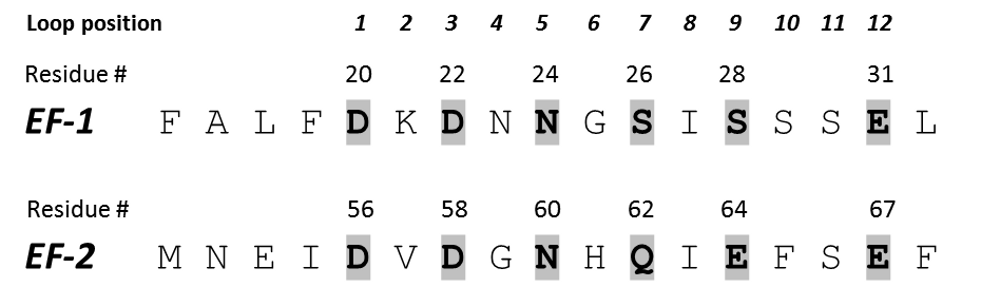

Supplement: Figure S1 — Sequence of Ca2+-binding loops in yCaM N-lobe. The 1–12 numbering corresponds to the amino acid positions within the linear sequence of the putative Ca2+-binding loops. Residues that provide Ca2+ ligands are shaded. (TIFF) [file pcbi.1003028.s001.tif]

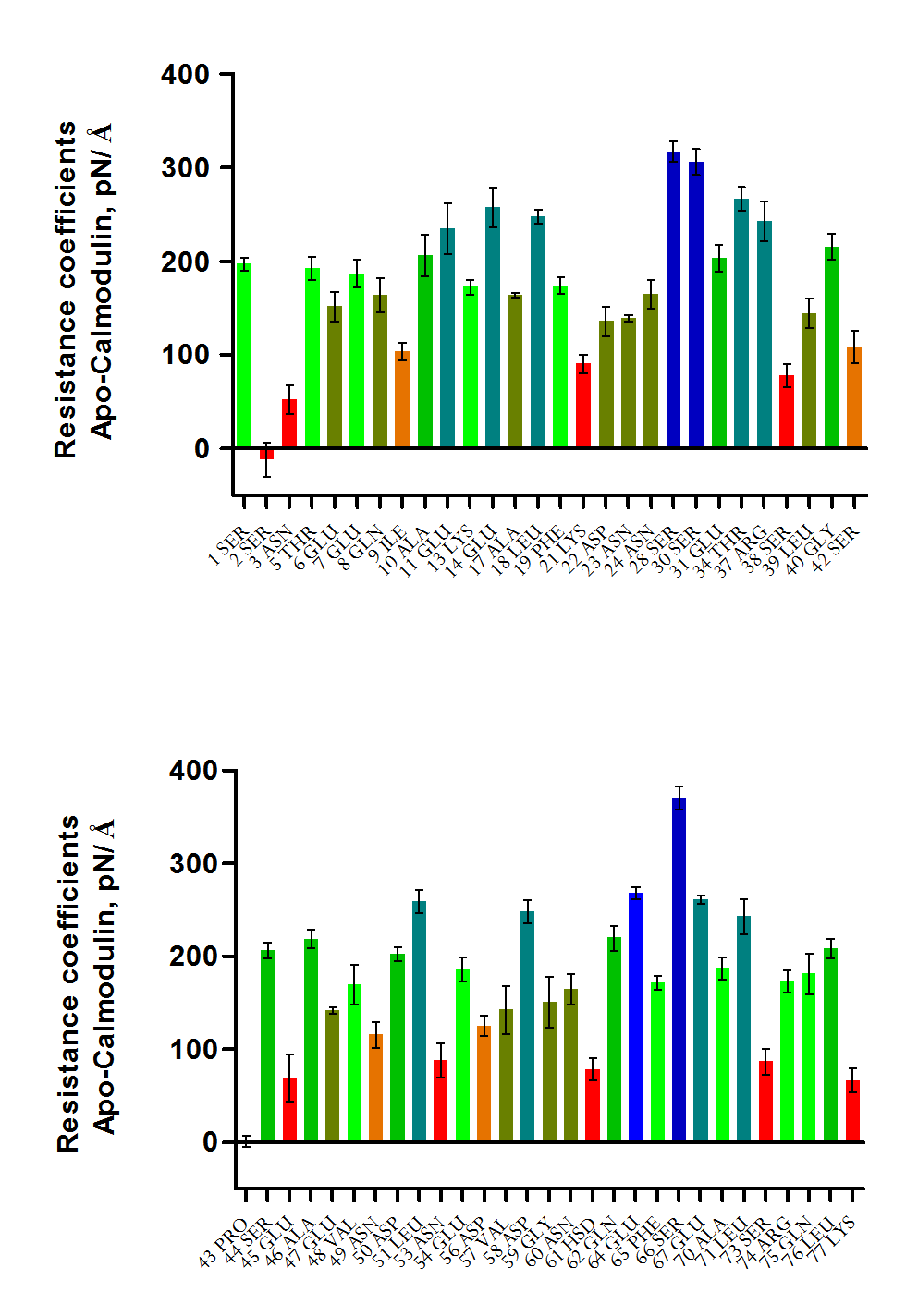

Supplement: Figure S2 — Resistance coefficients of surface-exposed residues in apo-yCaM. Surface-exposed residues in the structure of the apo-yCaM N-lobe (PDB: 1F54) were subjected to SMD analysis and the “resistance coefficients” calculated as described in the Methods. For each residue SMD analysis was carried out at least 12 times. The error bars represent the standard error of the mean. Color assignments are according the residues' “resistance coefficients” as described in the legend to Figure 2. (TIF) [file pcbi.1003028.s002.tif]

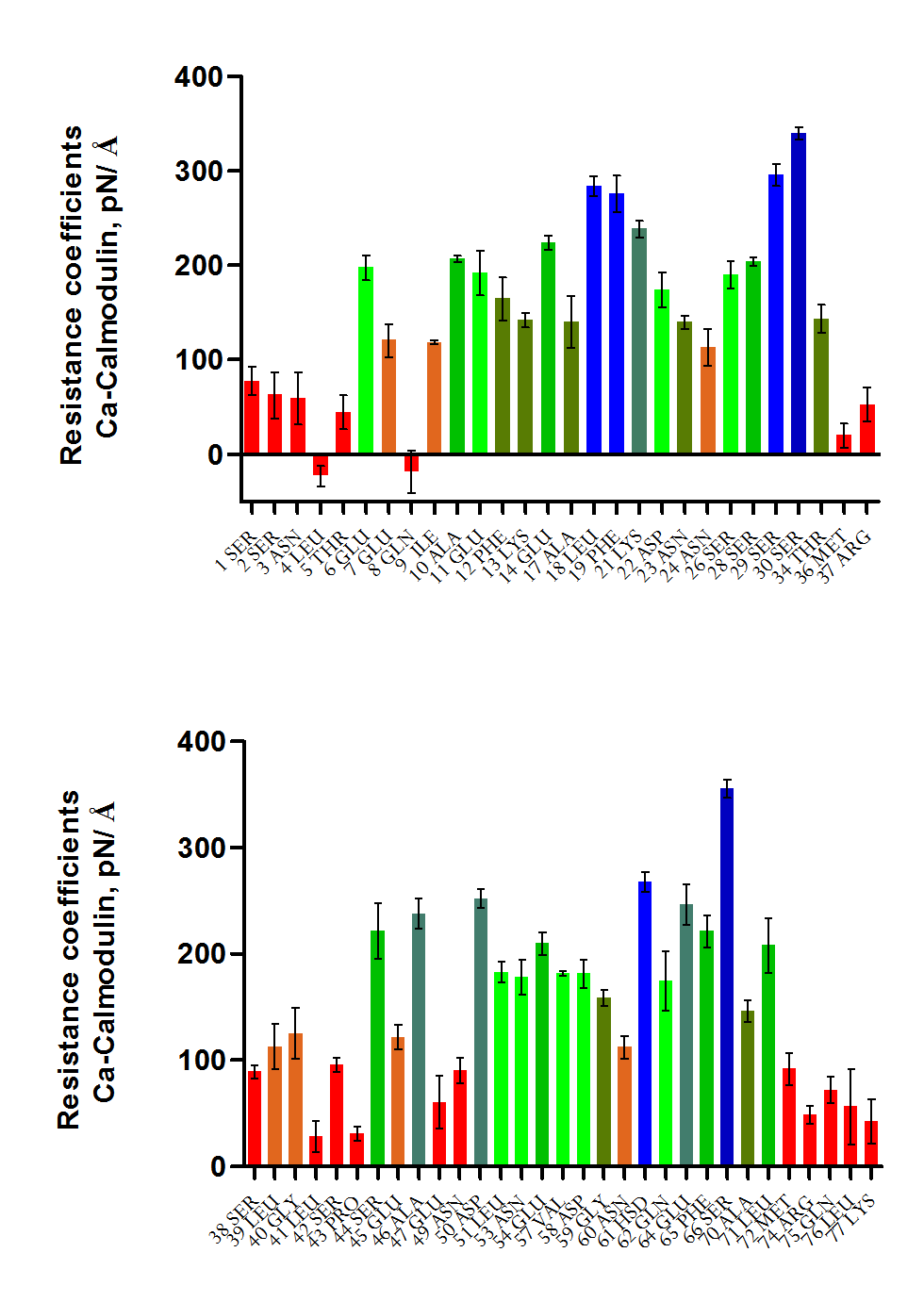

Supplement: Figure S3 — Resistance coefficients of surface-exposed residues in Ca2+-loaded yCaM. Surface-exposed residues in the structure of the Ca2+-loaded yCaM N-lobe (PDB: 1F55) were subjected to SMD analysis. Other details as in the legend to Supplementary Figure S2. (TIF) [file pcbi.1003028.s003.tif]

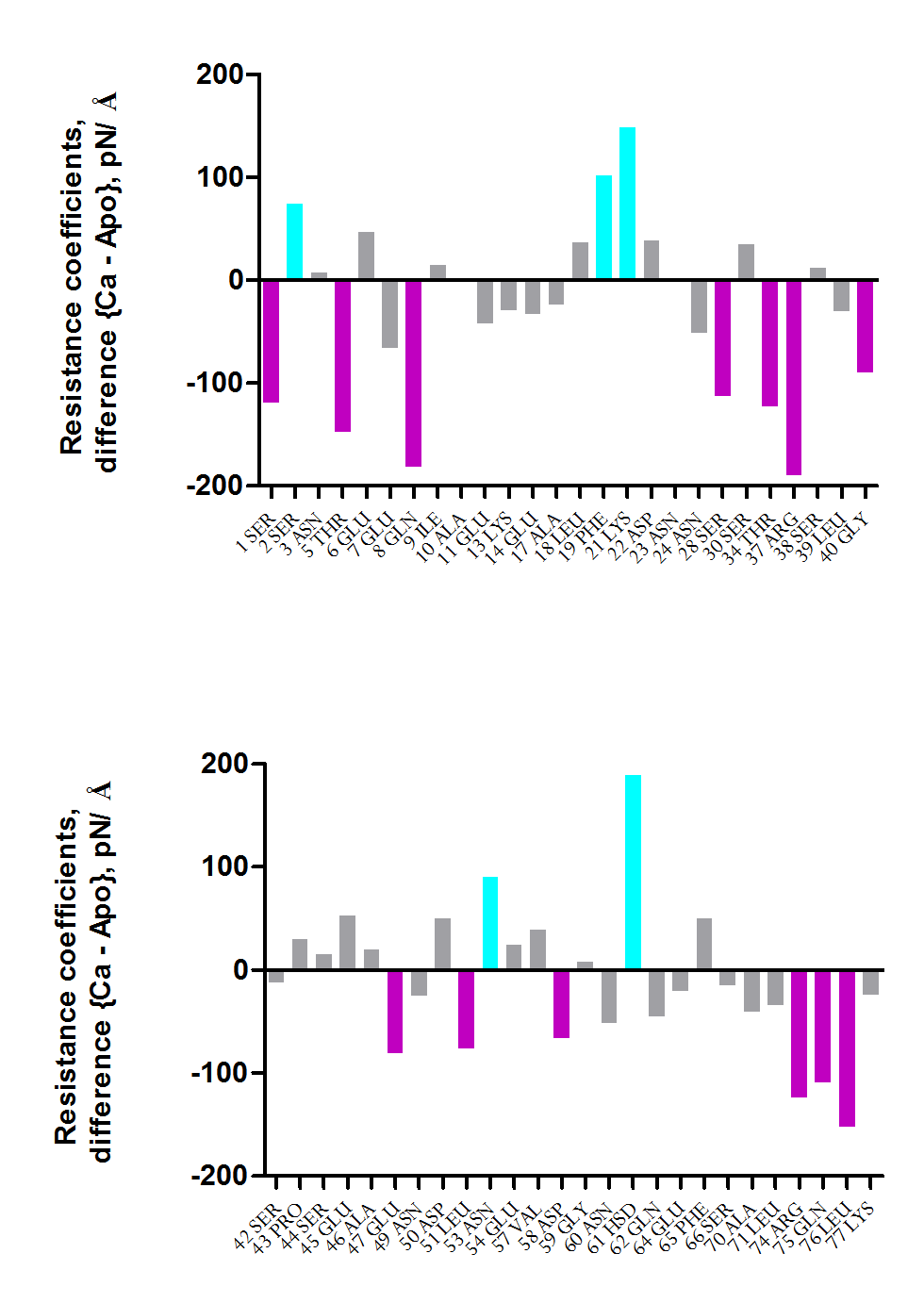

Supplement: Figure S4 — Ca2+-induced changes in resistance coefficients of surface-exposed residues. The “resistance coefficients” of surface-exposed residues in the structures of both apo- and Ca2+-loaded yCaM N-lobe were used to calculate the changes. Positive values correspond to residues whose stability increased as a result of Ca2+ binding, whereas negative values correspond to residues with increased mobility. Residues whose resistance coefficients increased or decreased by more than 70 pN/Å are colored in cyan or purple, respectively. In Figure 4 (bottom), the same residues are shown in the Surf representation and colored similarly. (TIF) [file pcbi.1003028.s004.tif]
